# Targeting EP4 downstream c‐Jun through ERK1/2‐mediated reduction of DNMT1 reveals novel mechanism of solamargine‐inhibited growth of lung cancer cells

**DOI:** 10.1111/jcmm.12958

**Published:** 2016-09-13

**Authors:** Yuqing Chen, Qing Tang, Qian Xiao, LiJun Yang, Swei S. Hann

**Affiliations:** ^1^ Laboratory of Tumor BiologyDepartment of Medical OncologyGuangdong Provincial Hospital of Chinese MedicineThe Second Clinical Medical CollageUniversity of Guangzhou Traditional Chinese MedicineGuangzhouChina

**Keywords:** human lung cancer cells, solamargine, EP4, ERK1/2, DNMT1, c‐Jun

## Abstract

Lung cancer is the most common cancer and the leading cause of cancer deaths worldwide. We previously showed that solamargine, one natural phytochemicals from traditional plants, inhibited the growth of lung cancer cells through inhibition of prostaglandin E2 (PGE_2_) receptor EP4. However, the potential downstream effectors of EP4 involving in the anti‐lung cancer effects of solamargine still remained to be determined. In this study, we further verified that solamargine inhibited growth of non‐small‐cell lung cancer (NSCLC) cells in multiple cell lines. Mechanistically, solamargine increased phosphorylation of ERK1/2. Moreover, solamargine inhibited the protein expression of DNA methyltransferase 1 (DNMT1) and c‐Jun, which were abrogated in cells treated with MEK/ERK1/2 inhibitor (PD98059) and transfected with exogenously expressed DNMT1 gene, respectively. Interestingly, overexpressed DNMT1 gene antagonized the effect of solamargine on c‐Jun protein expression. Intriguingly, overexpressed c‐Jun blocked solamargine‐inhibited lung cancer cell growth, and feedback resisted the solamargine‐induced phosphorylation of ERK1/2. A nude mouse xenograft model implanted with lung cancer cells *in vivo* confirmed the results *in vitro*. Collectively, our results show that solamargine inhibits the growth of human lung cancer cells through reduction of EP4 protein expression, followed by increasing ERK1/2 phosphorylation. This results in decrease in DNMT1 and c‐Jun protein expressions. The inter‐correlations between EP4, DNMT1 and c‐Jun and feedback regulation of ERK1/2 by c‐Jun contribute to the overall responses of solamargine in this process. This study uncovers an additional novel mechanism by which solamargine inhibits growth of human lung cancer cells.

## Introduction

Lung cancer is the most common cancer and the leading cause of cancer mortality worldwide 1. Most patients with lung cancer are diagnosed with an advanced, unresectable disease resulting in a poor prognosis. Among them, more than 80% of lung cancers are non‐small‐cell lung cancer (NSCLC) with little changes of low 5‐year survival rate 1. Although recent advances in understanding of the biological characteristics of this illness and multidisciplinary therapeutic approaches, such as individuated chemotherapy, targeted therapies, immune approaches and improved supportive care, have been reported 2, 3, the outcome remains dismal for patients with advanced disease. The choice of treatment for patients with advanced NSCLC still remains a significant challenge 4. This largely affects the quality of life and patient survival. Therefore, searching for more effective adjuvant strategies with maximizing efficacy and minimizing adverse effects is highly desired.

Natural compounds, such as solamargine (SM), the component of solanum lycocarpum fruit glycoalkaloid extract, demonstrated anti‐tumour properties in several cancer types 5, 6, 7, 8, 9. Early study found that combination of low concentrations of SM with low‐toxic topoisomerase II inhibitor epirubicin synergistically accelerated apoptotic cell death through up‐regulation of Fas expression and down‐regulated the expressions of HER2 and topoisomerase II alpha (TOP2A) in NSCLC A549 and H441 cells 10. Recently, one study showed that solamargine effectively inhibited the growth of melanoma cells, while minimum effects were observed in normal and benign cells, through triggering and disrupting both extrinsic and intrinsic apoptotic pathways 11. We previously demonstrated that solamargine inhibited the growth of NSCLC cells through inactivation of phosphatidylinositol 3‐kinase/Akt (PI3‐K/Akt) signalling pathway, followed by reduction of transcription factors, such as SP1 and p65, expressions. In turn, this resulted in the inhibition of prostaglandin E2 receptor E‐prostanoid receptor 4 (EP4) gene expression 12. However, the detailed mechanisms underlying this, especially the role of potential downstream effectors of EP4 in this process, still have not been well elucidated.

DNA methylation is the most common epigenetic modification in the mammalian genome. DNA methyltransferase 1(DNMT1), the major epigenetic enzyme, maintains the epigenetic state of DNA and genome stability by replicating CpG methylation signatures and producing heritable methylation patterns through cell metabolism 13, 14. Through the interactions with transcription factors, non‐coding RNAs, oncogenes and tumour suppressors, DNMT1 influenced cell survival, cell cycle arrest, senescence and cell death *via* methylation‐dependent and methylation‐independent pathways, which resulted in aberrant activation of the multiple downstream signals and controlled expression of genes, leading to cancer growth, progression and metastasis 15, 16, 17. Thus, approaches for inhibition of DNMT1 may become novel strategies for treating cancers 18, 19.

The transcription factor AP‐1 (activating protein‐1), a heterodimer of the c‐Jun and c‐Fos proteins, plays an important role in growth and metastasis of various tumours 20. As a member of the AP‐1 family of transcription activating complex and proto‐oncogene, overexpressed c‐Jun showed to significantly enhance cell growth and reduce apoptosis partly through regulation of AP‐1 targets and other pro‐invasion genes associated with resistance to anti‐cancer agents resulting in poor survival 21, 22, 23, 24. Thus, targeting c‐Jun could be potential for the prevention and treatment of cancer 23, 25.

The E‐prostanoid receptor 4 (EP4) subtype for prostaglandin E_2_ (PGE_2_), the family members of G protein‐coupled receptors, involves in a variety of biological functions, such as inflammation, allergy, parturition, tumorigenesis, growth and metastasis 26. Studies demonstrated that highly expression of EP4 has been found in several tumour types including lung and involved in development and progression of several cancer types 26, 27, 28, 29, 30. Thus, that targeting EP4 signalling demonstrated the therapeutic potential in the prevention and treatment of cancer 26, 27, 28, 29, 30, 31, 32. We previously demonstrated the critical role of EP4 expression in mediating the anti‐lung cancer effects of solamargine 12. As such, the functional role of EP4 and its downstream signalling in lung cancer onset and progression remain to be determined. While the information for the links of EP4 and c‐Jun in lung cancer development and progression has been reported 33, 34, the association between EP4, c‐Jun to DNMT1 remained largely unknown 35.

In this study, we further explored the potential mechanism by which solamargine inhibits growth of human lung cancer cells. Our results demonstrated that the DNMT1 and c‐Jun acted as the potential downstream effectors of EP4 in mediating the anti‐lung cancer responses of solamargine.

## Materials and methods

### Cell culture and chemicals

The human cancer lines H1650, H1975, PC9, A549 and H1299 were obtained from the Chinese Academy of Sciences Cell Bank of Type Culture Collection (Shanghai, China). All cell lines have been tested and authenticated for absence of Mycoplasma, genotypes, drug response and morphology. Cells were grown in RPMI 1640 medium (obtained from GIBCO, Life Technologies, Grand Island, NY, USA) with supplemented 10% foetal bovine serum. Lipofectamine 3000 reagent was purchased from Invitrogen (Shanghai, China). The polyclonal antibody against EP4 was obtained from Abcam (Cambridge, MA, USA). The antibodies against DNMT1, c‐Jun, the phosphor‐form (Thr202/204) of extracellular signal‐regulated kinases 1/2 (ERK1/2), and MEK/ERK1/2 inhibitor PD98059 were purchased from Cell Signaling Technology Inc (Beverly, MA, USA). Other chemicals unless indicated were obtained from Sigma‐Aldrich (St. Louis, MO, USA).

### Cell viability assay

Cell viability was measured using the 3‐(4, 5‐dimethylthiazol‐2‐yl)‐2, 5‐diphenyltetrazolium bromide (MTT) assay 36. Briefly, lung cancer cells were harvested and seeded in a 96‐well microtiter plate followed by treating with solamargine (6 μM) for up to 48 hrs. Afterwards, MTT solution (20 μl, 5 g/l) was added to each well, and cells were incubated at 37°C for an additional 4 hrs. Finally, the 200‐μl solvent dimethyl sulfoxide was added to each well for 10 min. The ELISA reader (Perkin Elmer, Victor X5, USA) was used to detect the Absorbance at 490 nm. Cell viability (%) was calculated as follows: (absorbance of test sample/absorbance of control) × 100%.

### Cell cycle analysis

This procedure was reported previously 12, 36. Briefly, NSCLC cells were cultured in 6‐well plates at 2 × 10^5^ cells/well and treated with increased doses of solamargine for 24 hrs. Afterwards, the cells were harvested, washed and resuspended in cold PBS and ethanol for 2 hrs at 4°C. The fixed cells were incubated in 1 ml of 0.1% sodium citrate containing propidium iodide (PI) RNase for 30 min at room temperature. The cell cycle distribution was detected by flow cytometry (FC500; Beckman Coulter, FL, USA), and the percentage of cells within the G0/G1, S, and G2/M phases were analysed using the MultiCycle AV DNA Analysis software (Phoenix Flow Systems, Inc., San Diego, CA, USA).

### Western blot analysis

The detailed procedure was reported previously 17, 36. In brief, equal amounts of protein from cell lysates were solubilized and separated on SDS polyacrylamide gels. Membranes were incubated with antibodies against EP4, DNMT1, c‐Jun, total and phosphor‐form (Thr202/204) of ERK1/2, followed by incubating with a secondary antibody raised against rabbit IgG conjugated to horseradish peroxidase (Cell Signaling Technology, Inc., Beverly, MA, USA). Afterwards, the membranes were visualized and enhanced chemiluminescence (Immobilon Western; Millipore, Billerica, MA, USA), followed by observing the signals under the Molecular Imager ChemiDoc XRS Gel Imagine System (Bio‐Rad, Hercules, CA, USA) and documenting the results.

### Transient transfection assay

This procedure was reported previously 12. The control or EP4, DNMT1 and c‐Jun overexpression constructs (pCMV6‐AC‐DNMT1, pCMV6‐AC‐c‐Jun, pCMV6‐AC‐EP4) were obtained from OriGene Technologies, Inc. (Rockville, MD, USA). Briefly, cells were seeded in 6‐well dishes and grown to 50–60% confluence. For each well, 2 μg of control, DNMT1, c‐Jun and EP4 plasmid DNA constructs were separately transfected into the cells using Lipofectamine 3000 reagent (Invitrogen, Shanghai, China) for up to 24 hrs based on the instruction from the provider, followed by treating with solamargine for an additional 24 or 48 hrs for other experiments.

### *In vivo* xenograft animal model

Experiments were performed according to the guidelines for the care and use of laboratory animals and were approved by the Animal Care and Use Committee of Guangdong Provincial Hospital of Chinese Medicine. A total of 33 eight‐week‐old female nude mice obtained from Guangdong Provincial Research Center for Laboratory Animal Medicine (Foshan, Guangdong, China) were maintained at the Animal Center of Guangdong Provincial Hospital of Chinese Medicine in a specific pathogen‐free environment with food and water provided. A549 cells carrying luciferase report gene (A549‐Luc, obtained from the Guangzhou Land Technology Co., Guangzhou, China) (1 × 10^6^ cells) in 100 μl PBS were injected subcutaneously in nude mice and allowed to grow for over 1 week when the initial measurement was made with calipers. Mice were randomly divided into control, low (4 mg/kg) and high doses (8 mg/kg) of solamargine group, which given *via* gavages (once another day) for up to 30 days (*n* = 11/group).

For bioluminescence imaging (BLI) procedure, mice were anesthetized by inhalation of 2% isoflurane. The D‐luciferin (150 mg/kg in approximately 100 μl; Caliper Life Sciences, Hopkinton, MA, USA) was injected into the peritoneal cavity of nude mice. The IVIS‐200 Imaging System (Xenogen/Caliper, Alameda, CA, USA) was used to measure the intensity of BLI signal. Tumour volume measurements were calculated using the formula for an oblong sphere: volume = (width^2^ × length). Quantification of bioluminescence was reported as photons/sec. The bodyweights of the mice were measured once a week. All mice were killed on day 30 in accordance with the Guide for the Care and Use of Laboratory Animals.

### Statistical analysis

All experiments were repeated a minimum of three times. Statistical analysis was performed with GraphPad Prism 5.0 software (GraphPad Software Inc, San Diego, CA, USA) with the use of an unpaired Student's *t*‐test (for comparison between two groups); a one‐way ANOVA with Tukey's multiple comparison tests. The results in graphs were presented as percentage of control. Asterisks shown in the figures indicate significant differences in experimental groups in comparison with the corresponding control condition. A value of *P* < 0.05 was regarded as statistically significant.

## Results

### Solamargine inhibited growth in multiple lung cancer cell lines and induced cell growth arrest in H1299 NSCLC cells

**Figure 1:**
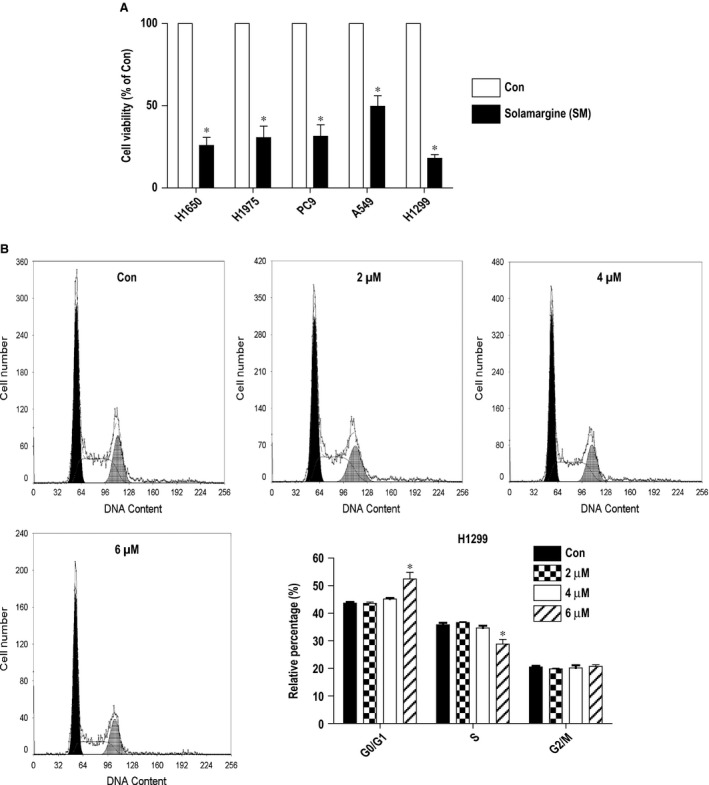
Solamargine inhibited growth in multiple lung cancer cell lines and induced cell growth arrest in H1299 cells. **(A)** Lung cancer cell lines (H1650, H1975, PC9, A549 and H1299) were treated with solamargine (6 μM) for up to 48 hrs. Afterwards, the cell viability was determined using the MTT assay as described in the Materials and methods section. **(B)** H1299 cells were treated with increased concentrations of solamargine for up to 48 hrs. Afterwards, the cells were collected and processed for analysis of cell cycle distribution by flow cytometry after propidium iodide (PI) staining. And the percentages of the cell population in each phase (G0/G1, S and G2/M) of cell cycle were assessed by Multicycle AV DNA Analysis Software. Data are expressed as a percentage of total cells. Values are given as the mean ± SD from three independent experiments performed in triplicate. *Significant difference as compared with the untreated control group (*P* < 0.05).

We previously showed that solamargine inhibited the growth of lung cancer cells 12. Herein, we found that solamargine (6 μM) inhibited growth in multiple NSCLC cell lines (Fig. 1A). By performing the cell cycle experiment, we also observed that, compared with the untreated control cells, solamargine significantly increased the proportion of cells at G0/G1 phase, while the proportion of cells at S phase was reduced at the 6 μM solamargine in H1299 NSCLC cells (Fig. 1B). These results again indicated the inhibitory property of solamargine for lung cancer cells.

### Solamargine increased the phosphorylation of ERK1/2

**Figure 2:**
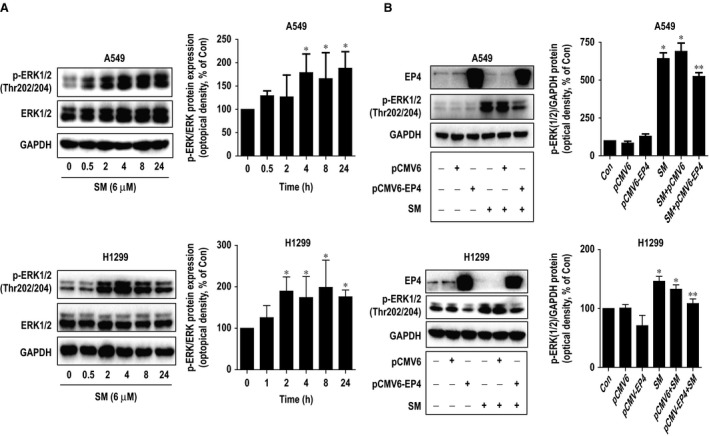
Solamargine increased the phosphorylation of ERK1/2. **(A**) H1299 and A549 cells were treated with solamargine (6 μM) in the indicated times, and cell lysate was harvested, and the expression of the phosphorylated and total protein of ERK1/2 was measured by Western blot analysis using corresponding antibodies. GAPDH was used as loading control. (**B)** A549 and H1299 cells were transfected with control and EP4 expression vectors for 24 hrs before exposing the cells to solamargine for an additional 24 hrs. Afterwards, the EP4 protein, phosphor‐ERK1/2 were determined using Western blot. GAPDH was used as internal control. Values in bar graphs were given as the mean ± SD from three independent experiments. *Significant difference compared with the untreated control group (*P* < 0.05). **Significant difference from solamargine treated alone (*P* < 0.05). ERK1/2, extracellular signal‐regulated kinases 1/2.

We next explored the signalling pathways that may be involved in the inhibitory effect by solamargine in lung cancer cells. We showed that solamargine increased the phosphorylation of ERK1/2 in a time‐dependent fashion with significant induction observed between 2 and 24 hrs in H1299 and A549 cells (Fig. 2A). Previously, we found that solamargine decreased the protein expression of EP4 in lung cancer cells 12. Intriguingly, herein, we observed that exogenously expressed EP4 significantly resisted the solamargine‐induced phosphorylation of ERK1/2 in H1299 and A549 cells (Fig. 2B). The above results indicated that solamargine reduced EP4 protein expression, followed by increasing the phosphorylation of ERK1/2 although more in‐depth experiments underlying this are still required to better elucidating this.

### Solamargine inhibited protein expression of DNMT1 through activation of ERK1/2

**Figure 3:**
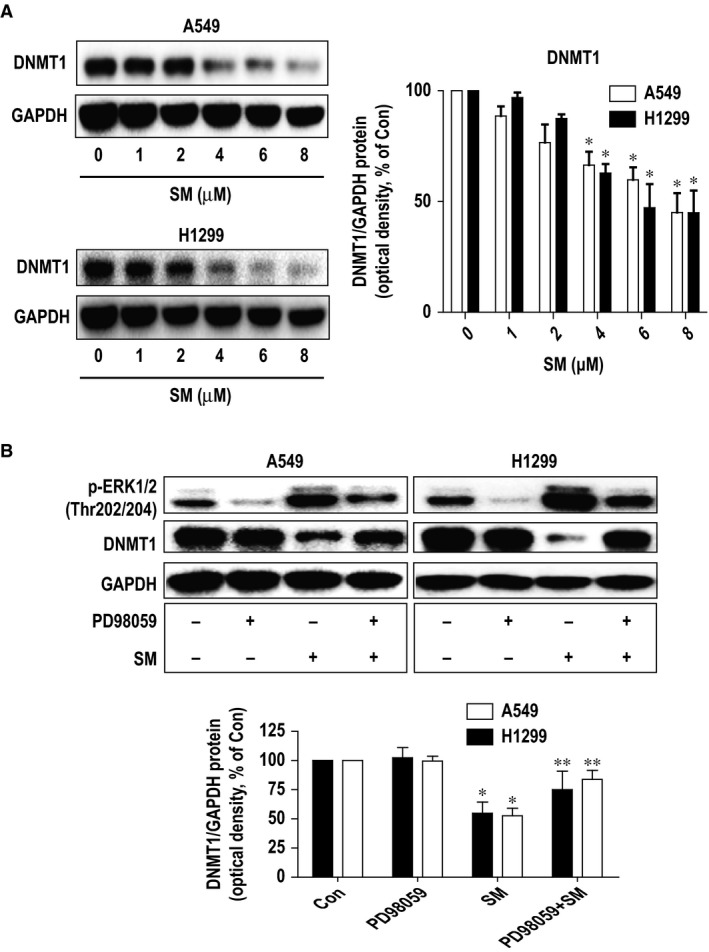
Solamargine inhibited protein expression of DNMT1 through activation of ERK1/2. **(A)** H1299 and A549 cells were exposed to increased concentration of solamargine for 24 hrs. Afterwards, the expression of DNMT1 proteins was detected by Western blot. **(B)** H1299 and A549 cells were treated with PD98059 (10 μM) for 2 hrs before exposing the cells to solamargine (6 μM) for an additional 24 hrs, followed by measuring the p‐ERK1/2 and DNMT1 protein expressions by Western blot. Values in bar graphs were given as the mean ± SD from three independent experiments. *Significant difference compared with the untreated control group (*P* < 0.05). **Significant difference from solamargine treated alone (*P* < 0.05). ERK1/2, extracellular signal‐regulated kinases 1/2.

Next, we further searched for potential molecular targets that mediated cell growth inhibitory effect of solamargine. Several lines of evidence have demonstrated that high expression of DNMT1 was found in several cancer types including NSCLC and that targeting of DNMT1 suppressed cancer cell growth 36, 37, 38. In this study, we showed that solamargine reduced the protein expression of DNMT1 in H1299 and A549 cells (Fig. 3A). Interestingly, the inhibitors of MEK/ERK1/2 (PD98059) significantly abrogated the effect of solamargine on DNMT1 protein expression suggesting that solamargine inhibited protein expression of DNMT1 through activation of ERK1/2 (Fig. 3B).

### Solamargine inhibited c‐Jun protein through inhibition of DNMT1 expression; exogenously expressed c‐Jun resisted the solamargine‐inhibited cell growth

**Figure 4:**
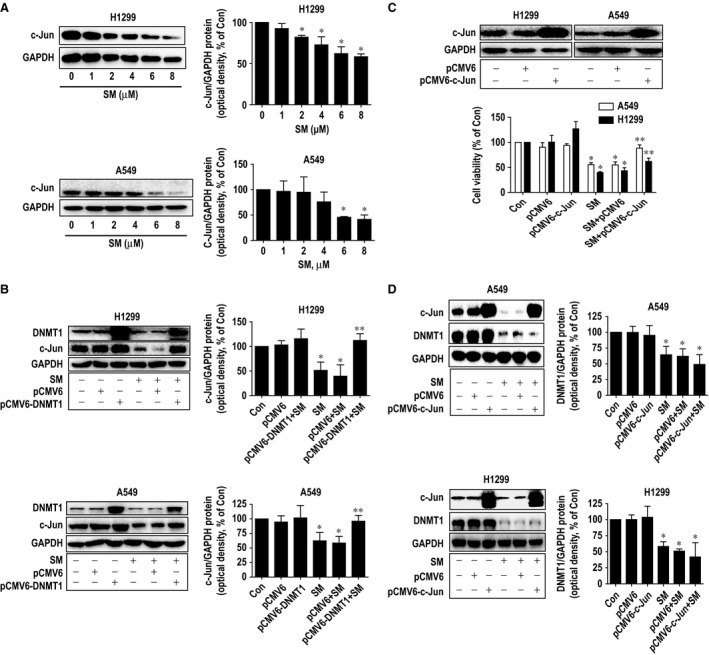
Solamargine inhibited c‐Jun protein through inhibition of DNMT1 expression; exogenously expressed c‐Jun resisted the solamargine‐inhibited cell growth. **(A)** A549 and H1299 cells were treated with increased concentration of solamargine for 24 hrs. Afterwards, the expression of c‐Jun protein was detected by Western blot. **(B)** A549 and H1299 cells were transfected with the control or expression construct of DNMT1 for 24 hrs before exposing the cells to solamargine (6 μM) for an additional 24 hrs. Afterwards, the expression of DNMT1 and c‐Jun proteins were determined by Western blot and was expressed as percentage of control in the mean ± SD of three separate experiments. GAPDH was used as internal control. **(C)** A549 and H1299 cells were transfected with the control or expression construct of c‐Jun for 24 hrs before exposing the cells to solamargine (6 μM) for an additional 24 hrs. Afterwards, the expression of c‐Jun protein and cell viability were determined by Western blot and MTT assays, respectively. GAPDH was used as internal control. Values in bar graphs were expressed as percentage of control in the mean ± SD of three separate experiments. **(D)** A549 and H1299 cells were transfected with the control or expression construct of c‐Jun for 24 hrs before exposing the cells to solamargine (6 μM) for an additional 24 hrs. Afterwards, the expression of DNMT1 and c‐Jun proteins were determined by Western blot and was expressed as percentage of control in the mean ± SD of three separate experiments. GAPDH was used as internal control. *Significant difference compared with the untreated control group (*P* < 0.05). **Significant difference from solamargine treated alone (*P* < 0.05).

Moreover, to gain insight into the molecular mechanism by which solamargine‐inhibited cell growth, we examined the potential downstream effectors of DNMT1. We showed that solamargine inhibited transcription factor c‐Jun protein expression, which was abrogated in cells transfected with exogenously expressed DNMT1 (Fig. 4A–B). Interestingly, exogenous expression of c‐Jun overcame the effect of solamargine on cell growth inhibition in H1299 and A549 cells (Fig. 4C). Note that overexpressed c‐Jun had no effect on solamargine‐reduced DNMT1 protein expression (Fig. 4D). Together, the above results suggested that c‐Jun acted as one of downstream effectors of DNMT1 and that inhibition of c‐Jun was involved in solamargine‐inhibited lung cancer cell growth.

### Overexpression of EP4 blocked the solamargine inhibited c‐Jun protein expression; exogenously expressed c‐Jun feedback reversed solamargine‐induced phosphorylation of ERK1/2

**Figure 5:**
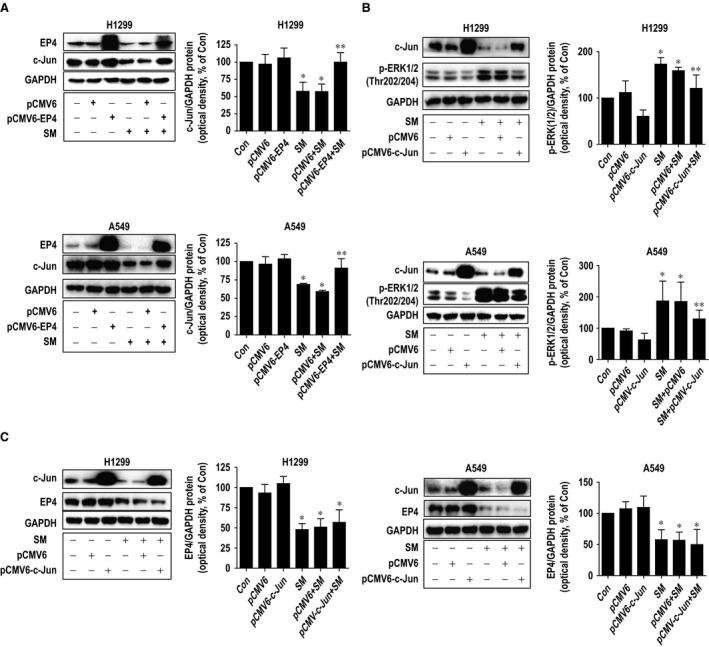
Overexpression of EP4 blocked the solamargine‐inhibited c‐Jun protein expression; exogenously expressed c‐Jun feedback reversed solamargine‐induced phosphorylation of ERK1/2. **(A)** A549 and H1299 cells were transfected with control and EP4 expression vectors for 24 hrs before exposing the cells to solamargine (6 μM) for an additional 24 hrs. Afterwards, the EP4 and c‐Jun proteins were determined using Western blot. GAPDH was used as internal control. **(B–C)** A549 and H1299 cells were transfected with the control or expression construct of c‐Jun for 24 hrs before exposing the cells to solamargine (6 μM) for an additional 24 hrs. Afterwards, the expressions of c‐Jun **(B–C)**, EP4 **(C)** and p‐ERK1/2 **(B)** were determined by Western blot. GAPDH was used as internal control. Values in bar graphs were given as the mean ± SD from three independent experiments. *Significant difference compared with the untreated control group (*P* < 0.05). **Significant difference from solamargine treated alone (*P* < 0.01). ERK1/2, extracellular signal‐regulated kinases 1/2.

To explore the possible functional relevance of EP4 expression changes following inhibition of c‐Jun by solamargine, we determined the ability of EP4 to regulate the c‐Jun expression. To this end, we showed that exogenously overexpressed EP4 blocked solamargine‐inhibited c‐Jun protein expression (Fig. 5A). Intriguingly, exogenously expressed c‐Jun feedback antagonized in part solamargine‐induced phosphorylation of ERK1/2 (Fig. 5B), while it had no effect on EP4 protein expression (Fig. 5C). These findings indicated that c‐Jun could be one of downstream effectors of EP4 and that a negative feedback regulatory loop of ERK1/2 by c‐Jun was occurred in this process.

### *In vivo* anti‐cancer activity in lung cancer cell is associated with reduction of EP4, DNMT1 and c‐Jun protein expressions and Induction of phosphorylation of ERK1/2

**Figure 6:**
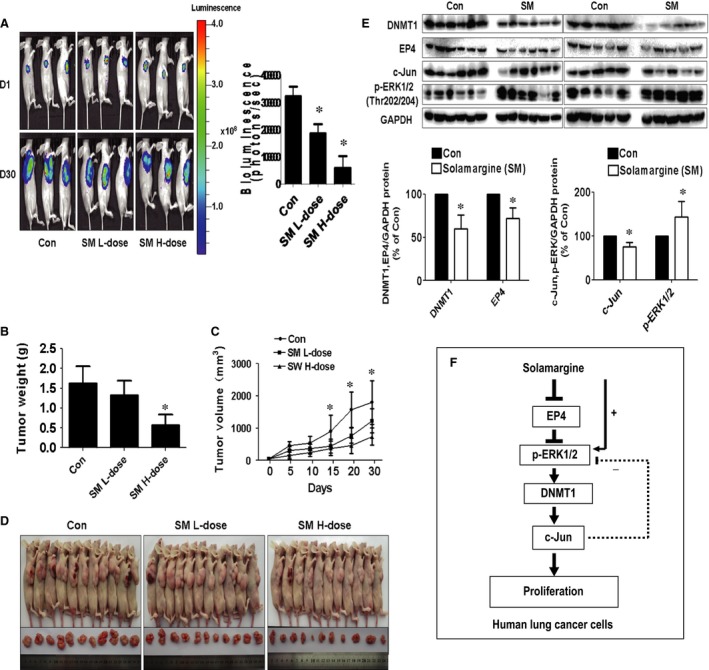
*In vivo* anti‐cancer activities in lung cancer cells are associated with reduction of EP4, DNMT1 and c‐Jun protein expressions and induction of phosphorylation of ERK1/2. Mice (*n* = 11/group) were divided to three groups [Con (saline), Low (L, 4 mg/kg) and High (H, 8 mg/kg) doses], and solamargine was given around the 7th day after tumour cells injection by gavages daily for up to 30 days. **(A)** The xenografts were assessed by *in vivo* bioluminescence imaging at the day 7 and the end of the experiments. The tumour growth was monitored by injecting luciferin in the mice followed by measuring bioluminescence using IVIS Imaging System. Quantification of signals was controlled by the acquisition and analysis software living image as described in Materials and methods section. Representative images are shown. (**B and C)** The xenografts were harvested, and the volume and weight of tumours were measured. **(D)** The photographs of control and solamargine‐treated xenografts and nude mice are shown. **(E)** At the end of the experiments, xenografted tumours were isolated from individual animals and the corresponding lysates were processed for detecting EP4, DNMT1, c‐Jun proteins and phosphor‐ERK1/2 by Western blot. GAPDH was used as loading control. Values in bar graphs were given as the mean ± SD from three independent experiments. *Significant difference from untreated control (*P* < 0.05). **(F)** The diagram shows that solamargine inhibits the growth of human lung cancer cells through reduction of EP4 protein expression and induction of phosphorylation of ERK1/2. This results in decrease in DNMT1 and c‐Jun protein expressions. The inter‐correlations between EP4, DNMT1 and c‐Jun, and negative feedback regulation of ERK1/2 by c‐Jun contribute to the overall responses of solamargine in this process. ERK1/2, extracellular signal‐regulated kinases 1/2.

We also tested the effect of solamargine in tumour growth and expressions of EP4, DNMT1 and c‐Jun in xenografted mouse model. We found that, compared with the control group, the high‐dose solamargine‐treated mice showed a significant delayed tumour growth, without any severe adverse events, as assessed by the Xenogen IVIS200 System (Fig. 6A). In addition, we noticed a significant reduction of the tumour weight and sizes in the high doses of solamargine treatment group as compared with the control group (Fig. 6B–D). By Western blot, fresh tumours harvested from the experiment showed that solamargine significantly decreased EP4, DNMT1 and c‐Jun protein expression and induced phosphorylation of ERK1/2 *in vivo* in the high‐dose solamargine treatment group compared with the control group (Fig. 6E).

## Discussion

Although improvements in early diagnosis and clinical treatment strategies have been made, the overall 5‐year survival for NSCLC patients still remains low 39, and the need to elucidate in‐depth mechanisms involved in the tumorigenesis of NSCLC and search for potential therapeutic targets are much required 1, 40. Thus, while studying and developing novel and new therapeutics to augment currently available treatment regiments with less adverse effects are important, further understanding of the molecular mechanisms of certain anti‐cancer agents for the treatment of malignancies such as lung cancer is also highly warranted.

We previously showed that solamargine, a steroidal alkaloid glycoside extracted from the traditional Chinese herb *Solanum incanum,* inhibited the growth of lung cancer cells through inactivation of PI3‐K/Akt signalling pathway, followed by reducing SP1 and p65 expression. In turn, this resulted in inhibition of prostaglandin E2 receptor EP4 gene expression 12. We previously found that EP4 overexpressing cells showed only a partial rescue from growth inhibition by solamargine 12, suggesting other additional players may be involved in this process. In this study, we provided an additional mechanistic evidence demonstrating that solamargine also affected the EP4 downstream effectors (DNMT1 and c‐Jun), thereby suppressing lung cancer cell growth. Our results suggest that activation of ERK played a role in this process. We reasoned that activation of this signalling pathway could be part of the anti‐tumour mechanism of solamargine although more studies are needed to confirm this. The activation of ERK axis has been reported to be involved in the anti‐tumour activities including lung cancer by different active compounds in several other studies although no studies showed the link of ERK signalling to the effects of solamargine 36, 41, 42, 43. On the contrary, opposite findings regarding the role of ERK signalling have been reported in other studies, such as the overall survival was significantly reduced in NSCLC patients with higher pERK1/2 expression 44, 45, 46, 47, 48. Thus, the possible dual roles of ERK in terms of tumour suppressor or tumour promoter have been depended on the activity of ERK signalling, feedback loops, interaction with other kinase signalling pathways, and different cell types studied 49.

Our results also implied that inhibition of EP4 expression was required in solamargine‐induced activation of ERK signalling, implying a possible upstream molecule of ERK signalling in this process. Regulation of PGE_2_ receptor subtypes, such as EP4 gene expression, has been shown to be involved in the influencing activation of several kinases including ERK in other studies demonstrating the complicated signalling networks that affected the tumour growth and progression, and the indispensable role of EP4 prostanoid receptor as a therapeutic target for the cancer treatment 50, 51, 52. Whether the effect of solamargine‐induced activation of ERK signalling was truly linked to EP4 expression or potential parallel signalling pathways (EP4 *versus* ERK) affected by solamargine existed still required to be determined.

Furthermore, our results suggested the critical role of DNMT1 in mediating the effect of solamargine on inhibition of lung cancer cell growth. Recent studies showed that, as critical epigenetic factors and tumour promoters, targeting of DNMT1 controlled the growth of several cancer cell types implying the important role of this molecule 15, 16, 53, 54. We believed that, as unfavourable tumour‐promoting role, DNMT1 could be a novel target in mediating the inhibitory effect of solamargine in lung cancer intervention. Moreover, we demonstrated a causative role of transcription factor c‐Jun that may involve in the effects of solamargine on lung cancer cell growth. Our findings suggested that c‐Jun may be one of downstream effecters of EP4 and DNMT1 and that inhibition of c‐Jun was required to mediate the effect of solamargine on lung cancer cell growth. The associations and links of EP4 and DNMT1 expressions to c‐Jun signalling have been shown in other studies 33, 34, 55. Expression of EP4 expression was involved in the PGE_2_‐induced oncogenic gene, such as phosphoinositide‐dependent kinase‐1 (PDK1) and α7 nicotinic acetylcholine receptor (nAChR), expression through inhibition of c‐Jun in bronchial epithelial and lung cancer cells 33, 34, 55. Nevertheless, more experiments are required to further elucidate the possible connections between EP4 and c‐Jun in this process. Of note, as the rescue effect by ectopic c‐Jun expression resistant to the growth inhibition by solamargine was only partial, we reasoned that potential molecules (e.g., STAT3, HER2, among others) other than c‐Jun may also be involved in the effect of solamargine‐inhibited cell growth 7, 56, which need to be determined.

In addition, we observed a novel feedback regulatory loop of ERK1/2 by c‐Jun. While the data of a direct feedback loop of ERK1/2 by c‐Jun are scarce, the negative feedback regulatory axis of ERK1/2 signalling pathway in influencing other gene expressions and subsequently cellular functions have been reported in other studies 57, 58, 59. Our results have demonstrated complicated regulatory signalling cascades that may involve in the overall anti‐cancer effects of solamargine in this process. Nevertheless, the detailed mechanism underlying this potential link in mediating the anti‐lung cancer effects of solamargine, or alternatively whether some parallel pathways have also occurred, still required to be elucidated in the future study.

We also observed the involvement of c‐Jun factor in mediating the response of solamargine‐inhibited lung cancer cell growth. The roles of c‐Jun signalling inter‐correlated with or without EP4 pathway in regulation of other gene expression, thereby influencing differentiation, angiogenesis, metastasis and invasion, have been shown in several cancer cell types 34, 60, 61, 62. Oncogenic transcription factor AP‐1 is critical for the proliferation of cancer cells, one study showed that histone deacetylase inhibitors (HDACIs), one of anticancer agents, targeted the AP‐1 c‐Jun/Fra‐1 dimer through transcriptional inhibition of mitogen‐activated protein kinase kinase 7 (MKK7) and RAF1 proto‐oncogene serine/threonine‐protein kinase (RAF1), this resulted in inhibition of growth in neuroblastoma cells 63. Another report showed that RNA‐binding protein tristetraprolin (TTP) inhibited cell proliferation *in vitro* and suppressed tumour growth *in vivo* through inhibiting c‐Jun expression and therefore increased Wee1 expression, a protein kinase and a key mammalian cell cycle regulator, and blocking NF‐κB/p65 nuclear translocation in breast cancer cells 64.

More importantly, our *in vivo* data were consistent with the findings from that *in vitro,* confirming the effect of solamargine on lung cancer growth inhibition and regulation of EP4 expression 12. The doses of solamargine used were based on our series of preliminary experiments *in vivo* and other study 65. In fact, there was scarce information available for the use of solamargine *in vivo*. We reasoned that more studies are required to confirm this. Moreover, additional studies are needed to further determine the critical role of EP4, DNMT1 and c‐Jun in this process using cells stable transfected with shRNAs and exogenous expression vectors of EP4, DNMT1 and c‐Jun genes in nude mice model.

In conclusion, our results show that solamargine inhibits the growth of human lung cancer cells through reduction of PGE_2_ receptor EP4 protein expression and induction of ERK1/2 signalling. This in turn results in decrease in DNMT1 and c‐Jun protein expressions. The inter‐correlations between EP4, DNMT1 and c‐Jun, and novel feedback regulation of ERK1/2 by c‐Jun contribute to the overall responses of solamargine in this process (Fig. 6F). This study uncovers an additional novel mechanism by which solamargine inhibits growth of NSCLC cells and suggests involvement of additional downstream signalling and targets of EP4 in lung cancer prevention and treatment.

## Conflicts of interest

The authors have declared that no competing interests exist.
